# Do statins increase and Mediterranean diet decrease the risk of breast cancer?

**DOI:** 10.1186/1741-7015-12-94

**Published:** 2014-06-05

**Authors:** Michel de Lorgeril, Patricia Salen

**Affiliations:** 1 Laboratoire TIMC-IMAG, CNRS UMR 5525, PRETA Cœur & Nutrition, and Faculté de Médecine, Université Joseph Fourier, Grenoble, France

**Keywords:** Cholesterol, Diabetes, Endocrine disruptors, Insulin resistance, Organic foods, Polyphenols, Statins

## Abstract

**Background:**

Physical exercise and healthy dietary habits are recommended to prevent breast cancer.

**Discussion:**

Increased intake of omega-3 fatty acids associated with decreased omega-6 - resulting in higher omega-3 to omega-6 ratio compared with Western-type diet - is inversely associated with breast cancer risk. The modernized Mediterranean diet with high omega-3 to omega-6 ratio, high fiber and polyphenol intake, and consumption of low-glycemic index foods reduces overall cancer risk and specifically breast cancer risk. It has been suggested that consuming no more than one alcoholic drink per day, preferably wine, is preferable. Eliminating environmental contaminants, including endocrine disruptors, and favoring organic foods to increase polyphenol intake and the omega-3 to omega-6 ratios were also shown to be beneficial. Cholesterol-lowering statins may decrease antitumor defenses; are toxic for the mitochondria; decrease the omega-3 to omega-6 ratio; increase body mass index, insulin resistance and diabetic risk; and have been associated with an increased breast cancer risk.

**Summary:**

Therefore, as well as making lifestyle changes to decrease breast cancer risk, we argue that physicians should carefully consider (and often avoid) therapies that may increase breast cancer or diabetes risk in high-risk women and women who wish to decrease their breast cancer risk.

## Background

Breast cancer (BC) remains a leading cause of death from cancer and a scientific challenge for the medical community [1]. One critical issue is how to implement an effective preventive strategy [2]. Risk factors such as genetic predisposition cannot be modified whereas other factors (unhealthy diet, sedentary lifestyle) can be avoided [3]. Other strategies - for instance, decreasing the length of time a woman’s breast tissue is exposed to estrogens - may help prevent BC but have proved difficult to implement [4].

Increasing protective factors is critical, in particular among high-risk women [3]. The effects of dietary factors have been examined. For instance, dietary fats have been extensively studied in the prevention of BC [5-7] but only marine omega-3 fatty acids (n-3) may be protective [5]. In a meta-analysis of 21 independent prospective cohort studies, a significant reduction of BC risk with marine n-3 was found [8]. By contrast, omega-6 fatty acids (n-6) may increase BC risk [9,10]. Although not all studies [9] show a link between n-6 and increased BC risk, the most recent and well-conducted studies actually indicate a positive association between n-6 and BC risk [10]. The pro-cancer effect of n-6 has also been suggested in randomized controlled trials in which n-6 intakes were modified [11,12]. These trials were not specifically referring to BC but to cancers in general, in particular because the numbers of cancers were too small to analyze specific cancers. However, in the same way as smoking increases the risk of lung, bladder and BC, the data suggest that n-6 may increase the risk of several cancers. If n-6 increase cancer risk in general, it is reasonable to think that they may also increase BC risk as epidemiological studies did suggest [10].

As both n-3 and n-6 may contribute to BC risk individually (but in opposite directions), they may introduce confusion in their respective analyses. Thus, when analyzing the associations between n-3 and BC risk, it is critical that n-6 is included in the analyses. This is what Yang *et al*. did in their recent study [13]. They used the ratio of n-3 to n-6 in a meta-analysis including 274,135 women from 11 independent prospective studies and found that women with a higher n-3/n-6 ratio had a significantly lower risk of BC compared to women with low n-3/n-6 ratio [13].

Thus, all the factors influencing the n-3/n-6 ratio are critical in BC risk [14]. Increased intake of n-3 and decreased intake of n-6 through consumption of foods rich in n-3 and poor in n-6 [10,15] - resulting in higher n-3/n-6 ratio - is therefore important to decrease BC risk [13,14]. Polyphenol flavonoids that increase marine n-3 by about 30% - possibly through the stimulation of endogenous synthesis - without altering n-6 levels [16-18] also result in a significant increase in the n-3/n-6 ratio. In fact, flavonoids are associated with a decreased BC risk [19,20].

Organic plant foods contain more polyphenols than similar conventional foods [21-24] and organic animal fat - for instance milk and milk products [25-27] - does have a higher n-3/n-6 ratio compared with conventional products. Thus, women who wish to decrease their BC risk may select organic plant and animal foods. Regarding food contaminants, a report from the American Institute of Medicine states that none of the potentially carcinogenetic contaminants, including organochlorine pesticides and polychlorinated biphenyls (PCBs), is linked to BC risk [28]. However, recent studies showing strong association between either estrogenic PCB congeners or dioxin and BC risk [29-31] do not confirm these optimistic conclusions. While further studies are needed, including studies of polymorphisms in the cytochrome P450 1A1 (CYP1A1) gene [32] (likely a confounding factor when studying the associations between PCBs and BC risk), these data are not reassuring. Regarding CYP1A1, this member of the CYP1 family participates in the metabolism of a vast number of xenobiotics including PCBs and dioxin. Four single nucleotide polymorphisms in *CYP1A1* have been studied concerning their potential implication on BC. A recent meta-analysis pointed to the A2455G G allele as a risk factor for BC among subjects of Caucasian origin [32]. Thus, further studies analyzing the relationships between estrogenic PCB congeners and BC risk should include *CYP1A1* polymorphisms as a potential marker of genetic predisposition to BC. In this context it is critical to recall that endocrine disruptors - such as phthalates - increase insulin resistance, diabetes and obesity [33-35], all of which increase BC risk (see below).

In the next section, we examine the critical importance of two major factors in breast cancer risk. One is protective (the modernized Mediterranean diet) whereas statins increase risk. The effects of both can be more easily understood at the light of the factors analyzed in the “Background” section.

## Discussion

### Statins and breast cancer risk

Other substances that influence both n-3/n-6 ratio and BC risk are the cholesterol-lowering statins. The effect of statins on cancer risk is a long story and still today there is no consensus [36-38]. The controversy began in 1996 with the publication of the Cholesterol and Recurrent Events (CARE) trial [39]. It was a double-blind randomized trial comparing the effects (versus placebo) of the cholesterol-lowering pravastatin against coronary event after myocardial infarction in 3,583 men and 576 women. Twelve out of 286 women in the statin group but only one out of 290 in the placebo group had BC at follow-up [39]. After that, most statin investigators took care not to include high-risk women in their trials [37] and carefully monitored them through repeated interim analyses for early detection of inter-group difference trends in cancer incidence. To further confuse the data, many statin trials were prematurely terminated - and it is likely that not all have been published - without valid scientific justification. Clearly, cancers diagnosed during drug trials are unlikely to be *ex nihilo* cancers and more likely to be dormant cancers clinically exposed by the treatment being investigated. As the process requires a minimal length of exposure, premature termination is the best way of avoiding the cancer issue in relation to any investigated drug. However, this process leads to confusion and prevents clarification of whether the investigated drug may increase cancer risk in the non-selected general population in whom the drug is then prescribed without precaution by unaware physicians. Despite this, a meta-analysis of clinical trials published in 2006 found a 33% increase in BC incidence with statins compared with a placebo [40]. It is noteworthy that confidence intervals were large (from 0.79 to 2.26) in that meta-analysis. However, there was great heterogeneity between trials (drug dosage, length of exposure) and curiously only five of the 26 randomized trials reported BC data [40], suggesting a striking lack of completeness of reporting of patient-relevant clinical trial outcomes, a well-known major source of bias and a substantial threat to the validity of clinical research findings [41]. In view of the inherent limitations of randomized trials discussed above, in particular premature termination and short follow-up, data from observational studies are critical to examine the statin-BC relationship.

In general, meta-analyses of observational studies reported no association between statin use and BC incidence. However, since high cholesterol may reduce cancer risk (see below), and as patients taking statins have spent most of their lives with high cholesterol - which is thought to lower cancer risk [37] - observational epidemiology is also facing difficulty in identifying statin cancer signals. In that context, even a lack of difference in BC risk between statin users and non-users in observational studies with long follow-up may suggest that statins increase BC risk. The recent demonstration that long-term (10-year) statin use was associated with a two-fold increase in BC risk among contemporary postmenopausal women [42] confirms the previous data suggesting that statins increase BC risk [36-40]. Regarding statin prescription and BC recurrence specifically, a Danish study suggested that one particular highly lipophilic statin (simvastatin) may be associated with a reduced risk [43]. However, as admitted by the authors, their study suffers major limitations. Briefly, the duration of exposure was short (a median of four years), the number of recurrences was small (n = 249 among statin users) and, very important, statin users and non-users were very different at baseline. This rendered adjustments for the many confounders - knowing that factors implicated in recurrence are not necessarily similar as those implicated in incidence - and between-group comparison very problematic. Still more important and admitted by the authors [43], confounding by indication likely explains their data [44] as the major indication for statin therapy is hypercholesterolemia, which is inherently associated with lower risk of BC recurrence [45].

The next question is whether there are biological explanations for the effect of statins on BC risk. First, statins interfere negatively with the metabolism of n-3 and n-6 - that is, they decrease the n-3/n-6 ratio [46-48] - which may in turn increase BC risk [13,14]. Second, statins lower cholesterol, and low cholesterol is often (but not always) associated with a high cancer rate [37]. Inconsistency in the cholesterol-cancer data is likely to reflect the existence of confounding factors. One of these factors could be insulin resistance or metabolic syndrome [49,50]. The Metabolic Syndrome and Cancer Project (Me-Can) - with more than 577,000 participants and a mean follow-up of 11.7 years - reported that cholesterol is negatively associated with BC risk, and this is a critical finding [50]. Third, a substance arising from cholesterol (dendrogenin A) is a key factor in the development of human BC [51], reinforcing the theory that high cholesterol may be protective. Fourth, statins are toxic to mitochondria [52,53], and mitochondrial dysfunction contributes to tumorigenesis and cancer progression [54,55]. Fifth, converging evidence supports the hypothesis that statins increase insulin resistance and new-onset diabetes, possibly (but not only) through mitochondrial toxicity in the muscles and other tissues [56-59]. This major side effect of statins was initially underestimated with regrettable consequences, some experts even stating that ‘the cardiovascular benefits of statin therapy exceed the diabetes hazard’ [60] while the trials upon which these claims were based were obviously flawed [61,62]. By contrast, studies indicate highly significant increases of incident diabetes among statin users [63,64], culminating in a 70% increase among postmenopausal women in the Women’s Health Initiative [65]. At the same time, it was learned that diabetes increases BC risk [66,67] as well as the overall risk of cancers and cancer death [68]. As diabetes is also a marker of long-standing insulin resistance - with chronically high insulin levels and high fasting blood glucose - it is critical that metabolic syndromes have also been associated with BC risk [69-72].

Recently, investigators curiously claimed that hypercholesterolemia is a risk factor for BC and that lowering circulating cholesterol levels (or interfering with its conversion to 27-hydroxycholesterol) may be a useful strategy to prevent and/or treat BC [73]. However, the effects of 27-hydroxycholesterol were tested in rather artificial cellular and animal models of BC and hypercholesterolemia [73]. Studies using more humanized models are required before these data could have any clinical impact. Finally, statins have been shown to increase the number of immune regulatory T cells, which in turn may hinder antitumor defenses and increase cancer risk [74].

Thus, statins may increase BC risk through increased insulin resistance and new-onset diabetes, decreased n-3/n-6 ratio, cholesterol lowering, mitochondrial toxicity and an immunomodulatory effect. Statin use also results in skeletal muscle toxicity and decreased physical activity [56-58]. For decreasing BC risk, reducing insulin resistance, metabolic syndromes and diabetes risk is beneficial, as shown with the Mediterranean diet in the next section. Additionally, international guidelines [1-3] recommend that women aim for optimal physical activity, which is known to decrease risk for both diabetes [75,76] and BC [1-4]. They also recommend that women should limit weight gain, especially around menopause, to reduce BC risk [1-3]. In that context, a recent report - 27,886 adults, 10-year follow-up - of a rapid increase in body mass index (equivalent to a 3- to 5-kg weight gain) among statin users compared with non-users is of concern [77]. Whatever the causes of that weight gain, be it reduced physical activity in relation with skeletal muscle toxicity [56-58], increased insulin resistance or increased caloric intake [77], it may contribute to the statin-induced increase in BC risk.

Regarding diabetes risk, increased fiber intake and consumption of flavonoids and n-3 are all inversely associated with diabetes risk [78-82]. In line with the fact that diabetes increases BC risk, not surprisingly fiber intake [83-86], flavonoids [19,20] and n-3 [8,10,13,14] are inversely associated with BC risk.

Finally, consumption of foods with a low glycemic impact - that is, foods with a low glycemic index (GI) - is associated with lower risks of diabetes [87,88] and BC [89-92].

### Modernized Mediterranean diet and breast cancer risk

The Mediterranean diet, the traditional dietary habits of people living around the Mediterranean Sea, is a well-known healthy dietary pattern [93]. A modernized version that includes traditional Mediterranean foods (for example, olive oil, non-refined wheat bread and wine) and foods not traditionally available to Mediterranean populations (for example, canola oil, margarines, low-fat dairy products) was tested in randomized trials and resulted in health benefits [93,94]. The combination of high fiber, high n-3/n-6 ratio, high polyphenols and low-GI foods represents a healthy dietary pattern. Adoption of such a healthy diet is clearly associated with a lower BC risk [95-99]. Among women with early-stage BC, increased adherence to a similar healthy dietary pattern was associated with decreasing risk of overall death and death from non-BC causes (*p* = 0.003) [100]. There was also a trend toward less BC death, the lack of statistical significance being explained by the quite small (n = 1,900) sample size and small number of BC deaths (n = 128) [100]. More specifically, increased adherence to the Mediterranean diet pattern is also clearly associated with fewer cancers [101], specifically pancreatic [102], gastric [103], colorectal [104], hepatocellular [105], prostate [106] and breast [107-109]. This is not unexpected since the Mediterranean diet increases the n-3/n-6 ratio on the one hand [10,93] and on the other decreases the risk of metabolic syndrome [110,111] and diabetes [112,113], both of which increase the risk of cancer - including BC - and cancer deaths [66-72]. Also, phenolic components of olive oil lowered body iron stores, which in turn may lower insulin resistance and metabolic syndrome [114]. Finally, the Mediterranean diet is an effective strategy for obtaining statistically and clinically significant weight loss [115-117], which in turn is considered a valuable strategy to reduce BC risk and improve survival after diagnosis [1-4].

The only limitation regarding the prevention of BC through adherence to the Mediterranean diet regards alcohol consumption. Moderate wine drinking is indeed a component of the traditional Mediterranean diet [93]. However, alcohol consumption increases BC risk [118], while the specific effect of wine is still unclear. The usual estimate for postmenopausal women who consume no more than one alcoholic drink per day is a 7% to 10% risk increase in comparison with non-drinkers [1,2]. This is small but significant. Alcohol consumption may also increase BC recurrence [119]. Women who use postmenopausal hormones should take particular care with BC risk in relation to alcohol consumption [1-4]. In some [120,121] but not all [122] studies, the excess BC risk with alcohol consumption is reduced by increasing the intake of folate. Accordingly, experts have stated that the Mediterranean way of drinking alcohol - regular and moderate consumption of polyphenol-rich wine mainly with folate-rich foods - does not appreciably influence the overall risk of cancer [123]. Given that moderate alcohol consumption also reduces the risk of cardiovascular disease [124], it appears that consuming approximately one alcoholic drink per day on average, including after BC diagnosis, is associated with optimal life expectancy without compromising BC-specific survival [125-127].

## Summary

Adhering to a healthy dietary pattern, specifically the modernized Mediterranean diet [93,94], should be the cornerstone of a lifestyle strategy to reduce BC risk in high-risk women and in women who wish to decrease their BC risk.

In the context of the Mediterranean diet, it is critical to increase plant and marine n-3 and decrease plant and animal n-6. High flavonoid consumption - which increases marine n-3 [16-18] - should be encouraged as it is associated with lower BC risk. To reduce insulin resistance and diabetes, which are associated with an increased BC risk, we argue that women should increase their consumption of fiber and favor low-GI foods. As far as possible, we feel that women should choose organic foods because of their effect on the n-3/n-6 ratio and because they contain fewer contaminants - and lower levels of each contaminant - in particular endocrine disruptor. Finally, we strongly argue that any drug thought to increase diabetes and/or BC risk - in particular, the statins and certain antihypertensive medications [128,129] - should be considered with a great deal of precaution and even prohibited in high-risk women. To lower blood pressure or to decrease the risk of cardiovascular disease, physicians do have alternative drugs and lifestyle strategies and it would be tragically unwise to persist in prescribing these specific anticholesterol and antihypertensive drugs in women wishing to decrease their BC risk.

National and international guidelines recommend healthy diet and physical activity to decrease BC risk [130]. We agree with this advice. It is time, however, to go further and be more specific. A specific dietary pattern such as the modernized Mediterranean diet, and not simply ‘consuming a diet rich in vegetables and fruits’, should be adopted to decrease BC risk. This is also an effective way of maintaining a healthy weight and preventing diabetes and cardiovascular disease. This also applies to BC survivors to prevent recurrence and improve survival [131,132].

## Abbreviations

BC: breast cancer; GI: glycemic index; n-3: omega-3 fatty acids; n-6: omega-6 fatty acids; PCB: polychlorinated biphenyls.

## Competing interests

The authors declare that they have no competing interests.

## Authors’ contributions

MdeL drafted the manuscript. PS critically revised the manuscript and gave final approval for publication. Both authors read and approved the final manuscript.
